# Contribution of dopaminergic polymorphisms to levodopa treatment response and drug concentration in Chinese patients with Parkinson’s disease

**DOI:** 10.1016/j.prdoa.2025.100333

**Published:** 2025-04-20

**Authors:** Xiaoqin He, Kai Shi, Chengjun Mo, Yi Zhang, Qin Xiao, Xiaodong Yang

**Affiliations:** a Department of Neurology and Institute of Neurology, Ruijin Hospital, Shanghai Jiao Tong University School of Medicine, Shanghai, China; b Geriatrics Health Care Ward, Qingdao Municipal Hospital, University of Health and Rehabilitation Sciences, Qingdao, Shandong, China

**Keywords:** Levodopa, Pharmacogenetic, Parkinson’s disease, Polymorphisms

## Abstract

**Background:**

Levodopa is the mainstay of treatments for Parkinson’s disease (PD), but large heterogeneity exists in patient response. Pharmacogenetic studies highlighted that genetic factors may play a relevant influence in this drug response variability.

**Objective:**

To explore the relationship between dopaminergic polymorphisms, levodopa treatment response, and drug concentration in Chinese patients with PD.

**Methods:**

Acute levodopa challenge test was conducted in 90 PD patients. Each patient underwent comprehensive neurological examination at baseline and after levodopa administration. Plasma levodopa concentrations were measured by liquid chromatography tandem mass spectrometry (LC-MS/MS) analysis. Twelve genetic polymorphisms in genes encoding dopaminergic enzymes (TH, DDC, COMT, MAOB and DBH) were genotyped.

**Results:**

Patients with the TH-rs6356 TT genotype showed higher ΔMDS-UPDRS-III scores compared to those with the CC + CT genotype after adjustment for the levodopa dose in the acute challenge test (P = 0.048). Furthermore, peak plasma levodopa concentration and Δplasma levodopa concentration were significantly higher in the TH-rs6356 TT group compared to the CC + CT group after adjustment (P = 0.007). Patients with the TH-rs6356 TT genotype exhibited a longer time to peak response compared to those with the CC + CT genotype (P = 0.042). However, this difference became non-significant after adjusting for levodopa dose (P = 0.066). The impact of other dopamine-related gene polymorphisms on levodopa efficacy appeared to be minimal in our study.

**Conclusions:**

Our preliminary results from a relatively small patients’ sample, may suggest that the rs6356 polymorphism in the TH gene could act as a possible modifier of levodopa response in PD.

## 1 Introduction

Parkinson’s disease (PD) is the second most common neurodegenerative disorder globally, affecting over 1 % of individuals aged 60 and older. According to the forecast, it is projected that by 2030, the prevalence of PD in China will escalate to approximately 4.94 million individuals, constituting half of the global PD patient population [1]. Clinically, PD manifests with both motor symptoms, including bradykinesia, rigidity, and resting tremor, as well as non-motor symptoms such as sleep disturbances, hallucinations, constipation, and cognitive impairment. The primary pathophysiological mechanism underlying these symptoms is the loss of dopamine neurons in the substantia nigra, making exogenous dopamine supplementation a cornerstone of PD treatment. [2].

Dopamine metabolism encompasses both anabolic and catabolic pathways. Dopamine biosynthesis begins with the conversion of L-tyrosine to levodopa, catalyzed by the enzyme tyrosine hydroxylase (TH) in the periphery. Levodopa, a precursor of dopamine, is able to cross the blood–brain barrier, where it is converted into dopamine. A significant portion of levodopa is metabolized into dopamine in the periphery by aromatic L-amino acid decarboxylase (AADC), which is encoded by the DDC gene. Further breakdown of dopamine into metabolites, including 3,4-dihydroxyphenylacetic acid (DOPAC), 3-methoxytyramine (3-MT) and norepinephrine in the central nervous system, is mediated by the enzymes catechol-O-methyltransferase (COMT), monoamine oxidase (MAO), and dopamine beta-hydroxylase (DBH) [3,4].

There are multiple pharmacological treatment options for PD with levodopa remaining the gold standard drug used in clinical practice to manage symptoms by replacing the dopamine lost. Although patients generally derive benefits from this symptomatic pharmacological management, the individual variability in response to levodopa poses a challenge to its practical utility in clinical settings. Despite long-term treatment, some patients continue to respond well to levodopa, whereas others fail to achieve the desired therapeutic effect [[5], [6], [7], [8]]. This variability in treatment response underscores the need to identify factors that influence levodopa efficacy. Given its multifactorial nature, this variability likely arises from both disease-related processes and clinical variables, as well as genetic factors. Recent progress has been made in identifying genetic biomarkers of drug response [9,10]. Efforts have also been made to define the role of genetic polymorphisms in optimizing pharmacotherapy for PD [11,12]. Pharmacogenetic studies have highlighted the role of genetic factors in shaping the variability of levodopa response, suggesting that consideration of pharmacogenetic could optimize therapeutic outcomes and facilitate personalized treatment strategies[13].

**Fig. 1:**
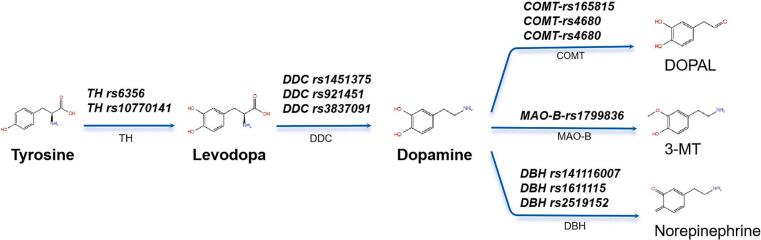
The SNPs in genes involved in the dopamine signaling pathway that were genotyped in the study.

Some studies have reported associations between polymorphic variations and levodopa response. For instance, the rs921451 and rs3837091 polymorphisms in the promoter region of the DDC gene could influence the motor response to levodopa [14]. In addition, polymorphisms in genes encoding dopamine degradation enzymes, such as rs4680 in the COMT gene and rs1799836 in the MAOB gene, have been implicated in regulating enzyme activity and the stability of dopaminergic signaling [15,16]. Although these genetic variations have been examined for their potential effects on motor response to levodopa, the results have been inconsistent, and the functional relevance of these variants remains uncertain. Moreover, ethnic differences among study populations may contribute to the observed discrepancies in findings [17,18]. Furthermore, while much previous research has focused on the genetic determinants of levodopa therapeutic outcomes, less attention has been given to the underlying changes in plasma levodopa concentration. To address this gap, our study investigates the associations between twelve genetic polymorphisms in genes encoding dopaminergic enzymes (TH, DDC, COMT, MAOB and DBH) (Fig. 1) and both plasma levodopa concentration and treatment response to an acute levodopa challenge in a relatively large cohort of Chinese PD patients.

## 2 Methods

### 2.1 Participants

A total of 90 PD patients were recruited from the Movement Disorder Clinic at the Department of Neurology, Ruijin Hospital, Shanghai Jiao Tong University School of Medicine. Participants were evaluated by at least two movement disorder specialists and met the United Kingdom Parkinson's Disease Society Brain Bank Clinical Diagnostic Criteria. Exclusion criteria included major organ dysfunction, other neurological diseases, and diabetes. Demographic and clinical data, including age, gender, body mass index (BMI), Hoehn & Yahr (H&Y) stage, age of onset, disease duration, were collected for each participant. The motor phenotype was classified into tremor-dominant (TD), postural instability and gait disorder dominant (PIGD) and indeterminate groups [19]. Levodopa equivalent dose (LED) was calculated using classic method [20]. All participants provided written informed consent, and the study protocol was approved by the Research Ethics Committee of Ruijin Hospital, Shanghai Jiao Tong University School of Medicine.

### 2.2 Levodopa challenge test (LCT)

A standardized Levodopa Challenge Test (LCT) was performed in the morning after a 36-hour withdrawal of dopaminergic agonists and overnight withdrawal of levodopa and other anti-PD medications. The levodopa challenge dose was set at 150 % of the participant’s regular morning LED. An immediate-release formulation of levodopa combined with 25 % benserazide (Roche) was administered. The “off-state” was defined as the period following at least 12 h without anti-PD drugs, while the best “on-state” was defined as the peak of levodopa benefit achieved during the LCT. Motor symptoms were assessed using the MDS-UPDRS-III in the “off-state” and repeated after levodopa administration at 30 min intervals for 5 h. Treatment response was measured by reduction in MDS-UPDRS-III score from the “off-state” to the best “on-state” condition (ΔMDS-UPDRS III). Latency to reach the best “on-state” was recorded.

### 2.3 DNA extraction

Venous blood samples (4–5 mL) were collected from participants on an empty stomach in the morning, mixed with EDTA to prevent coagulation. Genomic DNA was extracted from peripheral blood leukocytes (PBLs) using the standard phenol–chloroform extraction method. DNA samples were stored at − 80 °C until further use.

### 2.4 PCR and single nucleotide polymorphisms (SNP) genotyping

Primers for polymerase chain reaction (PCR) and single base extension were designed using Assay Design Suite V2.0 (Agena). The primers used for each SNP in the study are listed in Supplementary Table 1. SNP genotyping was performed by Shanghai Benegene Biotechnology Inc. using the MassARRAY system (Agena), based on matrix-assisted laser desorption ionization-time of flight mass spectrometry (MALDI-TOF) according to the manufacturer’s instructions. Briefly, DNA samples were diluted to 5–10 ng/µL, and 1 µL of DNA was combined with 0.95 µL of water, 0.625 µL of PCR buffer (containing 15 mM MgCl2), 1 µL of 2.5 mM dNTPs, 0.325 µL of 25 mM MgCl2, 1 µL of PCR primers, and 0.1 µL of 5 units/µL HotStar Taq polymerase (Qiagen). PCR amplification was carried out under the following conditions: 94 °C for 15 min, followed by 45 cycles of 94 °C for 20 s, 56 °C for 30 s, and 72 °C for 1 min, with a final incubation at 72 °C for 3 min. Post-PCR, unincorporated dNTPs were removed by shrimp alkaline phosphatase (Agena). The reaction was placed at 37 °C for 40 min, and the enzyme was deactivated by incubating at 85 °C for 5 min. After shrimp alkaline phosphatase treatment, the single primer extension over the SNP was combined with 0.755ul of water, 0.2 ul of 10X iPLEX buffer, 0.2 ul of termination mix, 0.041ul of iPLEX enzyme (Agena), 0.804ul of 10 uM extension primer. The single base extension reaction was carried out at 94 °C for 30 s and then 94 °C for 5 s, followed by 5 cycles of 52 °C for 5 s and 80 °C for 5 s, total 40 cycles, then 72 °C for 3 min. The reaction mix was desalted by adding 6 mg of cation exchange resin (Agena), mixed and resuspended in 25 ul of water. The completed genotyping reactions were spotted onto a 384 well spectroCHIP (Agena) using MassARRAY Nanodispenser (Agena) and determined by the matrix-assisted laser desorptionionization time-of-flight mass spectrometer. Genotype calling was analyzed using the MassARRAY Typer software version 4.0 (Agena).

### 2.5 Plasma levodopa concentration analysis

Blood samples were collected during the “off-state” condition and the best “on-state” condition following the levodopa challenge test, respectively. The samples were then centrifuged at 4 °C to obtain plasma, which was further stored at − 80°C until analysis. Plasma levodopa concentrations were measured using liquid chromatography-tandem mass spectrometry (LC-MS/MS) on a TSQ Vantage triple quadrupole mass spectrometer (Thermo Fisher Scientific, MA, USA). For the analysis, 200 µL of plasma was mixed with 30 µL of 10 µg/mL levodopa (RING-D3, 98 %; Cambridge Isotope Laboratories, MA, USA) as the internal standard. The mixture was vortexed for 1 min, followed by centrifugation at 15,000 × g for 20 min. The supernatant was analyzed on a Waters ACQUITY UPLC HSS T3 column (150 mm × 4.6 mm, 1.7 µm) at a flow rate of 0.2 mL/min at 4 °C. The mobile phase consisted of phase A (0.5 % formic acid in water) and phase B (methanol). Mass spectrometry was performed in positive ionization mode using selected reaction monitoring (SRM). Data were recorded and analyzed using Xcalibur software (version 4.0, Thermo Fisher Scientific).

### 2.6 Statistical analysis

Quantitative data are presented as mean ± standard deviation (SD) or median (interquartile range), while categorical data are expressed as frequency (percentage). The relationship between plasm levodopa concentration and MDS-UPDRS-III score was analyzed using Spearman rank correlation. Differences between groups were compared using the Mann-Whitney *U* test or chi-square test. Adjustments for the levodopa dose in the acute challenge were made using nonparametric analysis of covariance. The sample size was estimated using G*Power (version 3.1). An A priori power analysis was performed, specifying an alpha level of 0.05, power of 0.80, and an expected effect size (Cohen’s f = 0.25 ∼ 0.5). Based on these parameters, the required sample size per group was 46 to 180. Due to the location of the MAOB gene on the X chromosome, data were analyzed separately for men and women. Statistical analyses were performed using SPSS software (version 22.0; SPSS Inc., Chicago, IL) and R version 4.0.5. A significance threshold of 0.05 (2-tailed) was used for all analyses.

## 3 Results

### 3.1 Demographic features and genotype distributions of PD patients

A total of 90 idiopathic PD patients were included in the study. Clinical and epidemiological variables for all participants are presented in Table 1. Among the enrolled patients, 55.6 % were female, with a mean age of 67.1 ± 6.0 years and an average age of onset of 60.2 ± 7.3 years. The mean disease duration was 6.8 ± 4.0 years, and the average H&Y stage was 2.4 ± 0.6. The average levodopa dose used in the acute challenge was 276.6 ± 118.0 mg. After the levodopa challenge, the average change in MDS-UPDRS-III score (ΔMDS-UPDRS-III) was 16.4 ± 8.4, and the average change in plasma levodopa concentration (Δplasma levodopa concentration) was 210.0 ± 362.6 ng/mL. A positive correlation was found between ΔMDS-UPDRS-III and Δplasma levodopa concentration (R = 0.388, P < 0.001). The genotype and allele distributions of the studied genes are shown in Supplementary Table 2. The observed genotype and allele frequencies did not significantly differ from the expected frequencies according to Hardy-Weinberg equilibrium (P > 0.05).

**Table 1:** Characteristics of the patients in the study.

**Clinical Characteristics**	**Total PD patients N = 90**
Gender (male, n%)	40 (44.4)
Age (years)	67.1 ± 6.0
BMI (kg/m2)	25.2 ± 13.4
Age of onset (years)	60.2 ± 7.3
Disease duration (years)	6.8 ± 4.0
H-Y stage	2.4 ± 0.6
Phenotype (n, %)	
PIGD	54 (60.0)
indeterminate	9 (10.0)
TD	27 (30.0)
Wearing-off (n, %)	37 (41.1)
Dyskinesian (n, %)	10 (11.1)
Usual total levodopa daily dose equivalent (mg/day)	577.0 ± 300.3
Usual levodopa daily dose (mg/day)	412.5 ± 198.8
Levodopa dose in the acute challenge (mg)	276.6 ± 118.0

**Treatment response**	
“off-state” MDS-UPDRS Ⅲ	34.9 ± 12.8
best “on-state” MDS-UPDRS Ⅲ	18.5 ± 6.8
ΔMDS-UPDRS III	16.4 ± 8.4
Time to on (min)	49.3 ± 19.8
Time to peak (min)	80.2 ± 24.5
On duration (n, %)	
<150 min	41 (45.6)
≥150 min	49 (54.4)

**Drug concentration**	
Baseline plasma levodopa concerntration (ng/mL)	11.4 ± 8.3
best “on-state” plasma levodopa concerntration (ng/mL)	221.3 ± 364.4
Δplasm levodopa concentration (ng/mL)	210.0 ± 362.6

### 3.2 Association between dopamine synthetic enzyme polymorphisms and levodopa response

We first investigated the association between polymorphisms in two dopamine synthetic enzyme genes (TH and DDC) and levodopa response (Table 2). For TH-6356 and DDC-rs1451357 polymorphisms, no differences were observed in clinical features. Patients with the TH-rs10770141 GG and DDC-rs921451 TT genotypes required higher levodopa doses compared to those with the TH-rs10770141 AG and DDC-rs921451 CC + CT genotypes, respectively. Regarding treatment response and drug concentration parameters, patients with the TH-rs6356 TT genotype showed higher ΔMDS-UPDRS-III scores compared to those with the CC + CT genotype (P = 0.021). After adjustment for the levodopa dose in the acute challenge, this association was still significant (P = 0.048). Furthermore, peak plasma levodopa concentration and Δplasma levodopa concentration were significantly higher in the TH-rs6356 TT group compared to the CC + CT group (P = 0.001), and this difference remained significant after adjustment (P = 0.007). Patients with the TH-rs6356 TT genotype exhibited a longer time to peak response compared to those with the CC + CT genotype (P = 0.042). However, this difference became non-significant after adjusting for levodopa dose (P = 0.066). The peak plasma levodopa concentration (P = 0.042) and Δlevodopa concentration (P = 0.029) were also higher in the TH-rs10770141 GG group compared to the AG group; however, after adjustment for levodopa dose, no statistical difference was observed. The ΔMDS-UPDRS-III score was lower in patients with the DDC-rs921451 CC + CT genotype compared to the TT genotype (P = 0.030), but this difference was no longer significant after adjustment. No significant associations were found between levodopa response and the DDC-rs1451375 or rs3837091 variants.

**Table 2:** 

	**TH-rs6356**				**TH-rs10770141**				**DDC-rs1451375**				**DDC-rs921451**				**DDC-rs3837091**			
**Clinical Charactristics**	TT N = 52	CC + CT N = 38	P	P^a^	GG N = 78	AG N = 12	P	P^a^	CC + CA N = 67	AA N = 23	P	P^a^	CC + CT N = 65	TT N = 25	P	P^a^	AGAG.AGAG + AGAG.del N = 65	del.del N = 25	P	P^a^
Gender (male, n%)	25 (48.1)	15 (39.5)	0.417		37 (47.4)	3 (25.0)	0.145		29 (43.3)	11 (47.8)	0.705		30 (46.2)	10 (40.0)	0.599		31 (47.7)	9 (36.0)	0.317	
Age (years)	66.5 ± 6.1	67.9 ± 6.0	0.153		67.2 ± 6.1	66.3 ± 5.6	0.643		67.4 ± 5.4	66.0 ± 7.7	0.502		67.5 ± 5.7	66.1 ± 7.0	0.348		66.6 ± 5.2	68.2 ± 7.9	0.153	
BMI (kg/m^2^)	25.8 ± 17.5	24.4 ± 2.9	0.092		25.3 ± 14.4	24.1 ± 1.8	0.691		23.5 ± 3.1	29.9 ± 25.9	0.064		25.8 ± 15.7	23.6 ± 2.8	0.682		23.8 ± 3.0	28.7 ± 25.0	0.749	
Age of onset (years)	59.9 ± 7.2	60.7 ± 7.6	0.430		60.1 ± 7.5	61.0 ± 6.3	0.639		60.7 ± 6.9	58.8 ± 8.5	0.276		61.2 ± 7.1	57.9 ± 7.6	0.055		59.9 ± 6.7	61.2 ± 8.8	0.377	
Disease duraion (years)	6.6 ± 3.9	7.2 ± 4.0	0.443		7.1 ± 4.1	5.3 ± 2.3	0.215		6.7 ± 4.3	7.2 ± 3.0	0.253		6.3 ± 3.8	8.2 ± 4.2	0.047		6.8 ± 4.2	7.0 ± 3.3	0.361	
H-Y stage	2.4 ± 0.6	2.4 ± 0.5	0.640		2.4 ± 0.6	2.3 ± 0.6	0.577		2.4 ± 0.6	2.4 ± 0.5	0.387		2.3 ± 0.5	2.5 ± 0.6	0.289		2.4 ± 0.6	2.4 ± 0.6	0.583	
Phenotype (n, %)			0.151				0.289				0.237				0.300				0.011	
PIGD	33 (63.5)	21 (55.3)			49 (62.8)	5 (41.7)			37 (55.2)	17 (73.9)			42 (64.6)	12 (48.0)			33 (50.8)	21 (84.0)		
indeterminate	7 (13.5)	2 (5.3)			8 (10.3）	1 (8.3)			8 (11.9)	1 (4.3)			5 (7.7)	4 (16.0)			8 (12.3)	1 (4.0)		
TD	12 (23.1)	15 (39.5)			21 (26.9)	6 (50.0)			22 (32.8)	5 (21.7)			18 (27.7)	9 (36.0)			24 (36.9)	3 (12.0)		
Wearing-off (n, %)	25 (48.1)	12 (31.6)	0.116		35 (44.9)	2 (16.7)	0.125		24 (35.8)	13 (56.5)	0.082		22 (33.8)	15 (60.0)	0.024		26 (40.0)	11 (44.0)	0.730	
Dyskinesian (n, %)	6 (11.5)	4 (10.5)	1.000		9 (11.5)	1 (8.3)	1.000		5 (7.5)	5 (21.7)	0.135		6 (9.2)	4 (16.0)	0.589		5 (7.7)	5 (20.0)	0.197	
Usual total levodopa daily dose equivalent (mg/day)	609.8 ± 297.1	532.0 ± 302.8	0.107		605.9 ± 300.7	388.5 ± 228.2	0.014		564.0 ± 293.8	614.7 ± 322.1	0.326		534.9 ± 279.5	686.4 ± 329.8	0.016		581.1 ± 316.5	566.1 ± 258.9	0.752	
Usual levodopa daily dose (mg/day)	421.4 ± 178.3	400.4 ± 225.7	0.325		432.7 ± 199.5	281.3 ± 138.6	0.021		407.9 ± 201.3	426.1 ± 195.0	0.443		388.7 ± 173.7	474.5 ± 245.9	0.055		409.7 ± 213	420.0 ± 159.6	0.302	
Levodopa dose in the acute challenge (mg)	296.0 ± 120.1	250.0 ± 111.1	0.084		285.3 ± 115.5	219.8 ± 123.3	0.052		265.1 ± 112.8	310.3 ± 128.7	0.135		262.8 ± 117.0	312.4 ± 115.1	0.041		277.4 ± 118.8	274.5 ± 118.2	0.888	
**Treatment response**																				
“off-state” MDS-UPDRS Ⅲ	36.7 ± 12.8	32.4 ± 12.7	0.075	/	35.9 ± 13.1	28.1 ± 8.3	0.055	/	34.4 ± 12.1	36.3 ± 15.1	0.677	/	33.6 ± 13.2	38.2 ± 11.4	0.038	/	35.6 ± 12.4	33.1 ± 14.0	0.430	/
best “on-state” MDS-UPDRS Ⅲ	15.9 ± 6.6	22.1 ± 4.7	0.048	0.221	19.1 ± 6.9	14.8 ± 4.4	0.045	0.132	18.8 ± 7.1	17.7 ± 6.0	0.630	0.199	17.7 ± 6.3	20.7 ± 7.6	0.076	0.169	19.3 ± 6.6	16.5 ± 7.1	0.069	0.069
ΔMDS-UPDRS III	20.8 ± 8.2	10.3 ± 6.8	**0.021**	**0.048**	16.9 ± 8.7	13.2 ± 4.6	0.241	0.608	15.6 ± 6.4	18.6 ± 12.4	0.436	0.453	15.9 ± 9.2	17.6 ± 5.7	**0.030**	0.884	16.3 ± 7.7	16.6 ± 10.0	0.860	0.803
Time to on (min)	51.9 ± 21.5	45.8 ± 16.7	0.216	0.170	50.0 ± 20.3	45.0 ± 15.7	0.503	0.468	49.7 ± 19.2	48.3 ± 21.7	0.649	0.698	48.0 ± 20.4	52.8 ± 17.9	0.213	0.345	51.2 ± 19.6	44.4 ± 19.6	0.114	0.146
Time to peak	84.8 ± 27.7	73.8 ± 17.9	**0.042**	0.066	81.5 ± 24.6	71.3 ± 23.2	0.153	0.285	81.5 ± 25.5	76.3 ± 21.6	0.448	0.243	79.2 ± 26.2	82.8 ± 19.9	0.320	0.739	81.2 ± 26.9	77.4 ± 17.1	0.629	0.518
On duration (n, %)			0.768	0.905			0.771	0.634			0.473	0.566			0.512	0.623			0.854	0.845
<150 min	23 (44.2)	18 (47.4)			36 (46.2)	5 (41.7)			32 (47.8)	9 (39.1)			31 (47.7)	10 (40.0)			30 (46.2)	11 (44.0)		
≥150 min	29 (55.8)	20 (52.6)			42 (53.8)	7 (58.3)			35 (52.2)	14 (60.9)			34 (52.3)	15 (60.0)			35 (53.8)	14 (56.0)		
**Drug concentration**																				
Baseline plasma levodopa concerntration (ng/mL)	11.7 ± 8.6	10.9 ± 7.8	0.941	/	11.4 ± 8.5	11.2 ± 6.4	0.669	/	11.2 ± 8.7	11.8 ± 6.8	0.357	/	11.7 ± 8.3	10.4 ± 8.3	0.415	/	11.0 ± 8.3	12.4 ± 8.1	0.302	/
best “on-state” plasma levodopa concerntration (ng/mL)	320.6 ± 447.4	85.5 ± 103.9	**0.001**	**0.007**	238.9 ± 383	106.9 ± 177.1	**0.042**	0.488	197.5 ± 330.8	290.7 ± 449.8	0.708	0.524	252.4 ± 410.7	140.5 ± 182.5	0.875	0.056	219.2 ± 363.2	226.8 ± 375.1	0.777	0.903
Δplasm levodopa concentration (ng/mL)	308.9 ± 445.3	74.6 ± 102.0	**0.001**	**0.007**	227.5 ± 381.2	95.7 ± 174.7	**0.029**	0.484	186.3 ± 329.6	278.9 ± 446.5	0.778	0.524	240.7 ± 408.8	130.1 ± 181.3	0.975	0.058	208.2 ± 361.6	214.4 ± 372.7	0.784	0.916

Adjustments: Levodopa dose in the acute challenge.

### 3.3 Association between dopamine degradation enzyme polymorphisms and levodopa response

Next, we explored the association between polymorphisms in three dopamine degradation enzyme genes (COMT, MAO-B, DBH) and levodopa response (Table 3). No significant associations were found. However, patients carrying the COMT-rs165815 CT + CC genotype showed lower ΔMDS-UPDRS-III scores compared to those with the TT genotype, with a P value approaching statistical significance (P = 0.057) after adjusting for levodopa dose.

**Table 3:** 

Adjustments: Levodopa dose in the acute challenge.

## 4 Discussion

In the present study, we explored the associations between polymorphisms in genes encoding dopaminergic enzymes (TH, DDC, COMT, MAOB and DBH) and levodopa treatment response and plasma drug concentration in Chinese patients with PD. Our results indicate that the TH-rs6356 polymorphism may modulate the motor response to levodopa during an acute challenge in PD patients. Specifically, individuals carrying the TT genotype of TH-rs6356 exhibited an enhanced response to levodopa, accompanied by higher plasma levodopa concentrations compared to those with the CC or CT genotype. However, the impact of other dopamine-related gene polymorphisms on levodopa efficacy appeared to be minimal in our study.

Dopamine biosynthesis begins with the hydroxylation of L-tyrosine to levodopa by TH, which then undergoes decarboxylation by DDC to form dopamine. DBH can further convert DA to norepinephrine or package it into vesicles for release into the synapse in response to presynaptic action potentials. Once released, dopamine is either inactivated by re-uptake through the dopamine transporter or metabolized by COMT and MAOB. Given the involvement of these enzymes in dopamine synthesis and metabolism, genetic variations in these pathways may potentially influence the therapeutic efficacy of levodopa [21,22].

TH is the first and rate-limiting enzyme in the synthesis of catecholamines, including dopamine, noradrenaline and adrenaline. This enzyme is present mainly in the central nervous system. Its activity is tightly regulated through multiple mechanisms, including gene expression modulation via alternative splicing, post-transcriptional mRNA modifications, and enzyme phosphorylation. The TH gene is located at the telomeric end of chromosome 11p15.5. Mutations in TH have been linked to levodopa-responsive infantile Parkinsonism, and levodopa-responsive dystonia (DRD) [23,24]. Levodopa remains the primary treatment for these conditions, with most patients exhibiting a strong and sustained response. Among TH polymorphisms, rs6356, which causes a Val81Met substitution in the regulatory domain of the TH enzyme, has been the most well-characterized. SNP rs6356 is allele-specifc correlated with expression levels of the TH gene in the brain. The T allele of rs6356 is associated with higher TH gene expression and enhanced enzyme activity, and individuals with the TT genotype exhibit the highest expression levels, followed by CT and GG genotypes [25,26]. A recent study reports that rs6356 plays a protective role in late-onset PD, suggesting that the activity of the enzyme is important in aging [27]. In our study, we found that individuals carrying the TT genotype of TH-rs6356 showed an enhanced motor response to levodopa and higher plasma levodopa concentrations compared to those with the CC or CT genotypes. This suggests that upregulated TH expression, associated with the T allele, may lead to increased catecholamines synthesis and influence dopamine availability thereby enhancing the therapeutic response. Additionally, it is plausible that the TH rs6356 polymorphism could influence the response of central dopamine receptors, as higher dopamine levels may increase D2 receptor occupancy and enhance receptor activation, potentially altering the motor response to levodopa. This warrants further research to elucidate the underlying biological mechanisms [28].

To the best of our knowledge, this is the first study reported that the rs6356 polymorphism in the TH gene may act as a modifier of levodopa response in PD. The TH rs10770141 polymorphism entails a transition from the predominant C allele to the less common T allele in the promoter region. The T allele is associated with enhanced transcriptional activity [29]. TH rs10770141 modulates subjective effects of cocaine in participants with cocaine dependence. Homozygous individuals for the minor T allele account for differences in the subjective effects produced by cocaine [30]. However, we found no significant association between rs10770141 and levodopa response in our cohort.

DDC plays a crucial role in dopamine synthesis, converting levodopa into dopamine in the central nervous system and peripheral tissues. The role of DDC polymorphisms in modulating levodopa response in PD has received less attention in the literature. Some studies, such as those by Devos et al. and Li et al., have reported that DDC rs921451 T carriers exhibit a better response to levodopa [14,31], though these findings were not universally replicated. Instead, Moreau et al. found that rs921451 was not associated with the response to levodopa [32]. In our study, we observed that the ΔMDS-UPDRS-III scores were significantly lower in patients carrying the DDC-rs921451 CC + CT genotype than those with the TT genotype. However, this difference lost statistical significance after adjusting for levodopa dose. Additionally, the peak plasma levodopa concentration and Δlevodopa concentration was higher in the DDC-rs921451 TT genotype, although the results were only weakly statistically significant after adjustment. The variance in response may be due to differences in disease duration, levodopa dose, and other clinical factors, as patients with the TT genotype in our cohort had a longer disease duration and required higher levodopa doses. Ethnic differences and sample size may also contribute to the contradictory results. The lack of consistent findings across studies and the influence of clinical variables highlights the need for further research to clarify the role of DDC polymorphisms in levodopa response.

We also investigated the association between polymorphisms in three dopamine degradation enzyme genes (COMT, MAOB, and DBH) and levodopa response. The most extensively studied COMT polymorphism is rs4680, which regulates COMT enzyme activity. While some studies have found association between rs4680 and levodopa treatment response [33], our study did not observe any significant effects, which is consistent with the result from Lee et al [34]. The association between COMT rs165815 and rs6269 with levodopa response have never been studied in PD. rs165815 has already been studied in connection to treatment-resistant schizophrenia and rs6269 was reported associated with levodopa-induced dyskinesia in PD [35,36]. From our results, the analysis of rs6269 failed to show any association with acute response to levodopa, while we found a tendency for patients carrying the COMT-rs165815 TT genotype have a better motor response to levodopa. Larger-scale studies are needed to confirm these results. MAOB rs1799836 G allele polymorphism has been associated with an increased risk of developing levodopa-induced dyskinesia in PD [37]. One study found an increased risk of 2.84-fold for male individuals carrying the MAOB G allele to be treated with higher doses of levodopa [15]. However, no association between MAOB rs1799836 and levodopa response was observed in our study, consistent with previous research [31]. DBH catalyzes the conversion of dopamine to norepinephrine in the central nervous system and peripherally. DBH enzyme activity is modulated at the genetic level by the presence of several polymorphisms. Among these, rs141116007, rs1611115 and rs2519152 were investigated in several studies [17]. To the best of our knowledge, this is the first study examining the potential effect of DBH genetic polymorphisms on levodopa treatment response in PD patients. However, our study found no significant association between DBH genetic polymorphisms and levodopa response. These results align with findings from a study investigating the effect of DBH rs1611115 polymorphism on levodopa/carbidopa treatment for cocaine dependence, which also showed no impact [38].

Our study had several limitations. First, the relatively small sample size may have limited the statistical power to detect subtle associations. Second, a single levodopa challenge may not fully capture the long-term effects of levodopa therapy. Third, other factors might cause variation in responses to levodopa. Gastrointestinal disorders such as delayed gastric emptying, helicobacter pylori infection, constipation, small intestinal bacterial overgrowth, and gut dysbiosis can also alter levodopa pharmacokinetics, contributing to non-optimal responses [[39], [40], [41]]. Therefore, studies that combine genetic and microbiome data are needed to better understand the complex factors that contribute to individual differences in levodopa response. Moreover, most pharmacogenetic studies in PD to date have been cross-sectional or retrospective. Well-designed prospective studies are necessary to more accurately assess the impact of genetic factors on levodopa response.

## 5 Conclusion

In summary, our preliminary results from a relatively small patients' sample, may suggest that the rs6356 polymorphism in the TH gene could act as a possible modifier of levodopa response in PD. However, further validation in larger cohorts with more extensive genetic testing is required to confirm whether differences in TH could serve as predictive biomarkers for levodopa treatment efficacy in PD patients.

## 7 Contributions

XQ H, K S conceived the project and contributed equally to the work. CJ M and Y Z were responsible for clinical evaluation. XQ H and K S were responsible for Statistical analysis. XD Y, and Q X designed the study and oversaw the study. XD Y wrote the first draft of the manuscript, and all authors contributed to the revision and approved the final manuscript.

## CRediT authorship contribution statement

**Xiaoqin He:** Writing – original draft, Project administration, Methodology, Investigation, Formal analysis, Data curation. **Kai Shi:** Project administration, Methodology, Investigation, Data curation. **Chengjun Mo:** Project administration, Investigation. **Yi Zhang:** Project administration, Methodology, Data curation. **Qin Xiao:** Writing – review & editing, Supervision, Resources. **Xiaodong Yang:** Writing – original draft, Supervision, Resources, Conceptualization.

## Funding

This work was supported by the National Key R&D Program of China (Grant No. 2022YFE0210100), the Shanghai Rising-Star Program (Grant No. 22QA1405700), National Natural Science Foundation of China (Grant Nos. 82171246 and 82371251).

## Declaration of competing interest

The authors declare that they have no known competing financial interests or personal relationships that could have appeared to influence the work reported in this paper.
