# Supplementary material for: Contribution of dopaminergic polymorphisms to levodopa treatment response and drug concentration in Chinese patients with Parkinson’s disease
Source: Clin Park Relat Disord. 2025 Apr 20;12:100333. doi: 10.1016/j.prdoa.2025.100333 (PMC12264220; doi:10.1016/j.prdoa.2025.100333)
Supplement: Supplementary Data 1 [file mmc1.docx]

Table 1. Summary of the primers used for the analysis of the gene polymorphism.

| Gene | SNP | First PCR prime (5’-3’) | Second PCR prime (5’-3’) | UEP SEQ (5’-3’) |
| --- | --- | --- | --- | --- |
| TH | rs6356 | ACGTTGGATGTCAAACACCTTCACAGCTCG | ACGTTGGATGTGGCCTTTGAGGAGAAGGAG | AAGAGCAGGTTTAGCA |
|  | rs10770141 | ACGTTGGATGACTGCTAGCTCCTGGCTTC | ACGTTGGATGTCTCCAAGGGGAAGGCATCA | GGGTTCCTGTGGCCCCTTCTTT |
| DDC | rs921451 | ACGTTGGATGACACAATAAAGCCAGGGCAC | ACGTTGGATGACAAGAACACCCGGGTTTAC | GTCTTACACTTGTACCACTA |
|  | rs1451375 | ACGTTGGATGTGGGAGTGCATCATATTGGC | ACGTTGGATGTCTCCAAGGCTTATTAGCTG | GCATCATATTGGCCATAGAG |
| COMT | rs4680 | ACGTTGGATGTTTTCCAGGTCTGACAACGG | ACGTTGGATGCCAGCGGATGGTGGATTTC | TGCACACCTTGTCCTTCA |
|  | rs6269 | ACGTTGGATGTTGCTTGGAGTGCCACCATC | ACGTTGGATGTGACACGTCAGGCAACTGAG | CCCATCGCCCCCTTGTGTT |
|  | rs165815 | ACGTTGGATGTCTACAGACGGATACTCCAC | ACGTTGGATGTTTCAAGGGTTCCTTCTCAG | TGTACTCCACGGTGGACC |
| MAOB | rs1799836 | ACGTTGGATGTCTGGAATCTTCCCCATGGC | ACGTTGGATGTCCTTTAGGGAGCAGATTAG | ACTGGCAAATAGCAAAAG |
| DBH | rs1611115 | ACGTTGGATGGAGGGATCAAGCAGAATGTC | ACGTTGGATGTCAGTCTCACCACGGCACCT | GCCCTCAGTCTACTTG |
|  | rs2519152 | ACGTTGGATGGCGAAGCTGTGAGGAGTGA | ACGTTGGATGCCCTTGCGTCTGCCTCATC | GGAGTGAGCCCCTCG |
|  | rs141116007 | ACGTTGGATGTCAGGCACATGCACCTCC | ACGTTGGATGAGGGAAACCCTTTCTGGGTC | TGTGTGCACCTCCCCCCAT |

Table 2. SNP genotype distributions

| Gene | SNP | Major/Minor allele | Frequencies (n,%) | Genotype | Frequencies (n,%) |
| --- | --- | --- | --- | --- | --- |
| TH | rs6356 | C vs. T | 39:141 (21.7:78.3) | CC | 1 (1.1) |
|  |  |  |  | TC | 37 (41.1) |
|  |  |  |  | TT | 52 (57.8) |
|  | rs10770141 | A vs. G | 12:168 (0.7:93.3) | AA | 0 (0.0) |
|  |  |  |  | AG | 12 (13.3) |
|  |  |  |  | GG | 78 (86.7) |
| DDC | rs921451 | C vs. T | 88:92 (48.9:51.1) | CC | 23 (25.6) |
|  |  |  |  | CT | 42 (46.7) |
|  |  |  |  | TT | 25 (27.8) |
|  | rs1451375 | A vs. C | 88:92 (48.9:51.1) | AA | 23 (25.6) |
|  |  |  |  | AC | 42 (46.7) |
|  |  |  |  | CC | 25 (27.8) |
|  | rs3837091 | AGAG vs. del | 88:92 (48.9:51.1) | AGAG. AGAG | 23 (25.6) |
|  |  |  |  | AGAG. del | 42 (46.7) |
|  |  |  |  | del. del | 25 (27.8) |
| COMT | rs4680 | A vs. G | 50:130 (27.8:72.2) | AA | 6 (6.7) |
|  |  |  |  | AG | 38 (42.2) |
|  |  |  |  | GG | 46 (51.1) |
|  | rs6269 | A vs. G | 83:97  (46.1:53.9) | AA | 37 (41.1) |
|  |  |  |  | AG | 9 (10.0) |
|  |  |  |  | GG | 44 (48.9) |
|  | rs165815 | C vs. T | 89:91  (49.4:50.6) | CC | 21 (23.3) |
|  |  |  |  | CT | 47 (52.2) |
|  |  |  |  | TT | 22 (24.4) |
| MAOB | rs1799836 | C vs. T | 30:150 (16.7:83.3) | CC | 8 (8.9) |
|  |  |  |  | CT | 14 (15.6) |
|  |  |  |  | TT | 68 (75.6) |
| DBH | rs1611115 | C vs. T | 158:22  (87.8:12.2) | CC | 69 (76.7) |
|  |  |  |  | CT | 20 (22.2) |
|  |  |  |  | TT | 1 (1.1) |
|  | rs2519152 | C vs. T | 33:147 (18.3:81.7) | CC | 3 (3.3) |
|  |  |  |  | CT | 27 (30.0) |
|  |  |  |  | TT | 60 (66.7) |
|  | rs141116007 | I vs. del | 97:83  (53.9:46.1) | 1. I | 31 (34.4) |
|  |  |  |  | I. del | 35 (38.9) |
|  |  |  |  | del. del | 24 (26.7) |
